# Molecular-scale modeling of light emission by combustion: An *ab initio* study

**DOI:** 10.1038/s41598-019-49200-2

**Published:** 2019-09-03

**Authors:** Yoshiyuki Miyamoto, Tokutaro Komatsu

**Affiliations:** 1 Research Center for Computational Design of Advanced Functional Materials, National Institute of Advanced Industrial Science and Technology (AIST), Central 2, 1-1-1 Umezono, Tsukuba, Ibaraki 305-8568 Japan; 2 School of Medicine, Nihon University, 30-1, Oyaguchi-Kamicho, Itabashi-ku, Tokyo 173-8610 Japan

**Keywords:** Excited states, Density functional theory, Electronic structure, Atomic and molecular collision processes

## Abstract

Despite the advanced understanding of combustion, the mechanisms of subsequent light emission have not attracted much attention. In this work, we model the light emission as electronic excitation throughout the oxidation reaction. We examined the simple dynamics of the collision of an oxygen molecule (O_2_) with a kinetic energy of 4, 6, or 10 eV with a stationary target molecule (Mg_2_, SiH_4_ or CH_4_). Time-dependent density functional theory was used to monitor electronic excitation. For a collision between O_2_ and Mg_2_, the electronic excitation energy increased with the incident kinetic energy. In contrast, for a collision between O_2_ and SiH_4_ molecules, a substantial electronic excitation occurred only at an incident kinetic energy of 10 eV. The electronic excitation was qualitatively reproduced by analysis using complete active space self-consistent field method. On the other hand, collision between O_2_ and CH_4_ molecules shows reflection of these molecules indicating that small-mass molecules could show neither oxidation nor subsequent electronic excitation upon collision with an O_2_ molecule. We believe that this work provides a first step toward understanding the light-emission process during combustion.

## Introduction

Light emission through oxidation is a common phenomenon that is usually observed as combustion (burning). Combustion is understood as an oxidation chain reaction^1^, in which the emission of high thermal energy can be numerically expressed by solutions of the differential equation of the reaction diffusion model, namely, blowing up^2^ or a singular perturbation^3^. However, the reaction diffusion model cannot express light emission, which should be derived from excited electrons. Although several elemental reactions for the electronic excitation have been experimentally proposed^4^, no theoretical work regarding excited states has been reported so far. Large electron excitation has been reported with projectile kinetic energies on the order of kiloelectronvolts, and has been calculated by TDDFT Ehrenfest dynamics for the stopping power of hydrogen ions^5^ and the emission of secondary electrons by helium ions^6^ traversing a graphene sheet. However, in a kiloelectronvolt regime, the excitation mechanism is impact ionization rather than transition among several PESs close to each other. In this work, we identify molecular-scale oxidation processes that change the electronic system from its ground state to excited states. If the electronic system has higher energy than the ground state, the subsequent electronic transition to the ground state could cause light emission. To examine this scenario, we performed a molecular dynamics (MD) simulation assuming a high kinetic energy for an oxygen molecule (O_2_) colliding with a magnesium dimer (Mg_2_), a silane (SiH_4_) molecule, and a methane (CH_4_) molecule. Mg_2_ and SiH_4_ were selected as subjects because of their combustion reactions which are widely used in nanoparticle syntheses^7–10^ and film formation^8,11,12^. Meanwhile, CH_4_ was selected as a counterpart of SiH_4_ as C is the group IV elements like Si. Theoretical insights into excited states would contribute to precise control of these production processes. Several collision trajectories between the O_2_ molecule and the target molecule were examined by changing the kinetic energies of the incident O_2_ molecule from 4 to 10 eV with one initial atomic configuration.

We used two theoretical approaches. One was real-time time-dependent density functional theory (TDDFT)^13^ coupled with classical MD, which was used to monitor the potential-energy change upon collision of an O_2_ molecule and a target molecule. The monitored potential was compared with the potential energy surface (PES) obtained by static density functional theory (DFT) within the ΔSCF scheme^14^ along with a trajectory obtained with a real-time TDDFT-MD simulation. The other approach was the complete active space self-consistent field (CASSCF)^15^ method for computing the PESs of the same trajectory for the MD obtained by the real-time TDDFT simulations to validate the PESs obtained by the ΔSCF DFT scheme. The purpose of this work is to find electronic excitation at particular trajectory of MD. On the other hand, for further accuracy and direct comparison of light emission spectroscopy throughout combustion, stochastic analysis of multiple trajectories for O_2_ collision with a target molecule by changing the initial conditions is required. However, this analysis will be tackled in future work because it is too expensive with our current computational resources.

## Computational Schemes

The TDDFT calculations were performed within the local density approximation using the Perdew-Zunger functional^16^ fitted to the numerical result for the electron gas calculation^17^. The plane-wave basis set was used under the periodic boundary condition of an (*x*, *y*, *z*) box with a size of 10 × 10 × 20 Å^3^. To prepare the initial condition of the simulation, we used the following method described in ref.^18^. (1) An O_2_ molecule or a target molecule (Mg_2_, SiH_4_ or CH_4_) was located in each of the periodic vacuum boxes. The ground state of each molecule was computed separately by DFT. (2) The total valence charge densities of the O_2_ and target molecule in the cell were merged. A series of all the occupied molecular orbitals (MOs) of the O_2_ molecule and the target molecule were taken as set of wavefunctions of the initial condition. Next, we started the real-time propagation TDDFT calculation with initial kinetic energy of the O_2_ atoms of 4 to 10 eV within the Ehrenfest dynamics and monitored the TDDFT potential energy expressed as

1 $$\begin{array}{rcl}{E}_{{\rm{pot}}}^{{\rm{TDDFT}}}(t) & = & \sum _{{\rm{occp}}}[\int {\psi }_{{\rm{n}}}^{\ast }({\bf{r}},t)(-\frac{1}{2}{\rm{\Delta }}){\psi }_{{\rm{n}}}({\bf{r}},t)d{\bf{r}}\\  &  & +\iint {\psi }_{{\rm{n}}}^{\ast }({\bf{r}},t){v}_{{\rm{nl}}}({\bf{r}},{\bf{r}}^{\prime} ,t){\psi }_{{\rm{n}}}({\bf{r}}^{\prime} ,t)d{\bf{r}}d{\bf{r}}^{\prime} ]+\frac{1}{2}\iint \frac{\rho ({\bf{r}},t)\rho ({\bf{r}}^{\prime} ,t)}{|{\bf{r}}-{\bf{r}}^{\prime} |}d{\bf{r}}d{\bf{r}}^{\prime} \\  &  & +\int {E}_{{\rm{X}}{\rm{C}}}[\rho ({\bf{r}},t)]d{\bf{r}}+\sum _{{\rm{I}}}{Z}_{{\rm{I}}}\int \frac{\rho ({\bf{r}},t)}{|{\bf{r}}-{{\bf{R}}}_{{\rm{I}}}(t)|}d{\bf{r}}+\sum _{{\rm{I}}\ne {\rm{J}}}\frac{{Z}_{{\rm{I}}}{Z}_{{\rm{J}}}}{|{{\bf{R}}}_{{\rm{I}}}(t)-{{\bf{R}}}_{{\rm{J}}}(t)|}.\end{array}$$

In Eq. (1), *ψ*_n_(**r**, *t*) is the time-dependent Kohn-Sham orbital^13^ and the electron density, *ρ*(**r**, *t*), consists of the sum of the norm of the occupied Kohn-Sham orbitals. The first and the second terms on the right-hand side of Eq. (1) are electron kinetic energy and the contribution of the sum of the non-local part of the pseudopotentials. We used Troullier-Martins norm-conserving pseudopotentials^19^. The third and fourth terms are the Hartree and exchange-correlation energies of electrons, respectively, and the last two terms are Coulomb potential for electron-ion attraction and ion-ion repulsion, respectively. We adopted the adiabatic exchange-correlation functional for *E*_XC_[*ρ*(**r**, *t*)]. The Eq. (1) was computed in momentum space using the formalisms of ref.^20^, which was also used to compute the forces on all ions used for the Ehrenfest MD simulation. All the valence wavefunctions were expressed by using the plane-wave basis set with a cutoff energy of 61 Ry. In performing the TDDFT-Ehrenfest simulation, we used the spin-unpolarized approximation. Within this approximation, the triplet ground state of the O_2_ molecule is mimicked by assigning half occupation for each of the doubly degenerate O_2_ π* MOs.

The real-time propagation of the Kohn-Sham orbitals was computed by solving the time-dependent Kohn-Sham equation^13^ as

2 $$i\frac{\partial {\psi }_{{\rm{n}}}({\bf{r}},t)}{\partial t}={H}_{KS}[\rho ({\bf{r}},t)]{\psi }_{{\rm{n}}}({\bf{r}},t).$$

In equation (2), *H*_*KS*_[*ρ*(**r**, *t*)] is the Kohn-Sham Hamiltonian given by a functional derivative of Eq. (1) with respect to ${\psi }_{{\rm{n}}}^{\ast }({\bf{r}},t)$. For the numerical solution to equation (2), we employed the fourth-order split-operator method^21,22^ and used the code FPSEID^23^. The interval of the time-step 0.726 atsecond was used for time-evolution of both electrons and ions that warrants the energy conservation with error less than 1 × 10^−5^ eV per O_2_ and Mg_2_. The same precision holds for the case of O_2_ and SiH_4_, and the case of O_2_ and CH_4_.

There should be several ways to monitor electronic excitation; The one is taking projections of the time-dependent Kohn-Sham orbitals obtained from Eq. (2) to the occupied and empty static Kohn-Sham orbitals on the same atomic positions. This method may approximately work when electronic charge density between the ground and excited states does not differ so much, and thus the Kohn-Sham Hamiltonian between the ground and excited states can be regarded as common. Meanwhile, like current oxidation cases, the reaction is followed by significant charge transfer that modifies the Kohn-Sham Hamiltonian. Therefore, orbital projection to those obtained by different Hamiltonians is rather vague to judge excitation. An alternative way to monitor electronic excitation is just to compare potential energies obtained by TDDFT-Ehrenfest dynamics and those by static DFT, which was preferred in this work. To analyze PESs within the DFT scheme, we performed ΔSCF calculations with snapshots of atomic coordinates along with the trajectory of the TDDFT Ehrenfest dynamics under several orbital occupation patterns. The detailed settings are described in later. The computed PESs should be validated by other formalisms. We employed the CASSCF scheme using several snapshots of a trajectory obtained with the current TDDFT Ehrenfest dynamics. The Firefly^24^ code based on GAMESS^25^ was used with the modified 6-31G basis set where the Mg 3*p* orbitals were omitted. For Mg_2_ + O_2_, the O 2 *s* and 2*p* orbitals and the Mg 3 *s* orbital were taken as the active space with 16 electrons. The geometry of a triplet excited state that corresponds to 2Mg^+^ + ${{\rm{O}}}_{2}^{2-}$ (charge-transfer [CT] state) was optimized. For this calculation, O 2*p* orbitals and Mg 3 *s* orbitals with 12 electrons were considered as the active space. In addition, the active space for SiH_4_ + O_2_ consisted of O 2 *s* and 2*p* orbitals and Si 3 *s* and 3*p* orbitals. Overall, 16 orbitals with 20 electrons were considered. Excited states with spin multiplicity up to 5 were considered. For the case of CH_4_ + O_2_, we did not perform CASSCF calculations.

## Results and Discussion

We describe three cases for an O_2_ molecule colliding with a Mg_2_ molecule, a SiH_4_ molecule, and a CH_4_ molecule. For all cases, the Cartesian coordinates of the initial position of the center of mass of O_2_ were (0, 0, 10 Å), where the *z*-axis was set as the direction of the incident velocity of the O_2_ molecule. The center of mass of the target molecule was set at the origin. The molecular orientation of the O_2_ molecule was set to along the direction with Cartesian coordinates of (1, 1, 1), whereas the molecular axis of the Mg_2_ molecule was in the (1, 0, 0) direction. For the initial orientation of the SiH_4_ molecules, two H atoms were set in the *zx* plane with common negative *z* values, and the other two H atoms were in the *yz* plane with a common positive *z* value, placing the Si atom at the origin. The geometries before the collision are visualized in the snapshots of the TDDFT-Ehrenfest dynamics which will appear later. For the case of O_2_ collision to a CH_4_ molecule, the initial orientation of the CH_4_ was set as *x* and *y* components of C-H bonds are parallelt to (1, 1), (−1, −1), (1, −1), and (−1, 1) directions, as indicated in snapshots of the TDDFT-Ehrenfest dynamics which will appear later.

### O_2_ collision with Mg_2_

**Figure 1:**
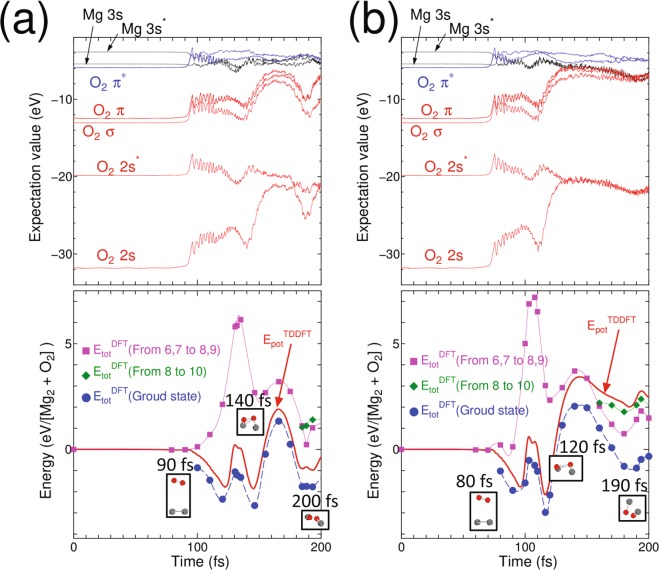
(**a**) (Top)Time evolution of the expectation values of molecular orbitals (MOs) for O_2_ colliding to Mg_2_ with incident kinetic energy 6 eV. Note that the alternation of the expectation values between Mg_2_ MOs and those of O_2_ MOs occurred. (Bottom) Time-variation of the potential expressed as Eq. (1) for O_2_ collision to Mg_2_, obtained by real-time TDDFT Ehrenfest dynamics, and by DFT along with snapshots within the ΔSCF scheme. In this panel, red solid lines denote the results of TDDFT Ehrenfest dynamics. Meanwhile, the blue circles are for DFT ground states. The green diamonds are obtained by the ΔSCF calculation promoting one electron from 8th MO to 10th MO. Meanwhile, purple squares are obtained by promoting two electrons from 6th and 7th MOs to 8th and 9th MOs. Small insets are snapshots of atomic coordinates for O atoms (red ball) and Mg atoms (gray balls), respectively. (**b**) Same as (**a**) but with incident kinetic energy of 10 eV.

In performing the TDDFT Ehrenfest dynamics, the incident kinetic energies of the O_2_ molecule were 4, 6, and 10 eV. Figure 1(a) shows the results for an incident kinetic energy of 6 eV and (b) shows the results for 10 eV. The results for an incident kinetic energy of 4 eV are not shown. The top panels show the time evolution of the expectation values of the Kohn-Sham Hamiltonian with respect to each MO of O_2_ and Mg_2_. There are nine occupied MOs per spin; seven MOs belong to the O_2_ molecule with two of them half-occupied, and two MOs belong to the Mg_2_ molecule. The energy levels of the fully occupied MOs of the Mg_2_ molecule with the initial geometry are higher than the half-occupied MO of the O_2_ π^*^ molecule (sixth and seventh orbitals in increasing order of the expectation values in Fig. 1(a)), which are doubly degenerate. Because of this ordering in expectation values among these MOs, the ground state DFT calculation should cause charge transfer from the Mg_2_ molecule to the O_2_ molecule, even when these molecules are separated. The TDDFT Ehrenfest dynamics can naturally express charge transfer, which occurs only when these molecules are closer^18^. The top panels in Fig. 1(a,b) show the alternation of the expectation values between the Mg_2_ and O_2_ MOs. But we must note here that the alternation does not rigorously mean the electronic excitation which should be expressed by comparing potential energy between TDDFT and static DFT as mentioned above. The bottom panels show the time evolution of the potential energy throughout the real-time TDDFT Ehrenfest dynamics. We also performed the DFT calculation for the excited state within the ΔSCF scheme; we considered up to 10 Kohn-Sham orbitals to evaluate one of the excited-state PESs. Later than 100 fs, the top two expectation values of the fully occupied MOs, originating from the Mg_2_ molecule, alternated to those of two half-occupied MOs, originating from the O_2_ molecule. Therefore, we re-labeled the eighth and ninth MOs as half-occupied and the sixth and seventh MOs as fully occupied. The plot of the ΔSCF potential-energy calculations was computed for the electronic ground state with the eighth and ninth MOs half-occupied. In contrast, the excited states were computed by assuming the promotion of one electron from the eighth to the ninth MO (green diamonds in Fig. 1(a,b)), and by assuming the promotion of two electrons from the sixth and seventh MOs to the eighth and ninth MOs, (purple squares in Fig. 1(a,b)).

**Figure 2:**
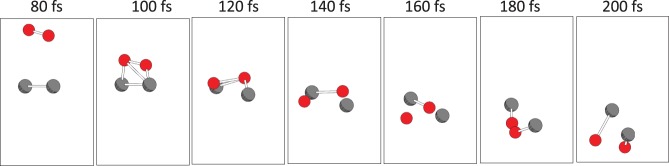
Snapshots of O_2_ colliding to Mg_2_ with incident kinetic energy of 10 eV. Red balls are O atoms, gray balls are Mg atoms.

With an incident kinetic energy of 4 eV, the simulation showed tentative Mg–O bond formation, which disappeared after O–O bond recovery throughout the simulation. Thus, we concluded that with the current initial position, a kinetic energy of 4 eV is not enough for oxidation. On the other hands, for incident kinetic energies of 6 and 10 eV, Mg–O bond formation was observed (insets in Fig. 1(a,b)). Next, we focus on the potential energies and compare them with the PESs obtained by the ΔSCF calculation within DFT. For an incident kinetic energy of 6 eV, the time evolution of the potential energy obtained by TDDFT was finally located slightly above the PES of the ground state, but this was far below the lowest excited PESs obtained by the ΔSCF calculation. This result may correspond to the PES mixing discussed in previous works^26,27^. As shown in the insets in the bottom of Fig. 1(a), two Mg–O bonds were eventually formed. When the incident kinetic energy was increased to 10 eV, the TDDFT potential-energy plot was in a higher position (bottom of Fig. 1(b)). (Note that the snapshots of the TDDFT-Ehrenfest dynamics is shown in Fig. 2). The TDDFT potential was close to the excited state promoting one electron from the eighth to the tenth MO, at which the formation of Mg-O was observed again. The current simulation assumed that only the projectile O_2_ molecule had an incident kinetic energy and the target Mg_2_ was still. Classical kinetics tells us that about 40% of the net collision energy should be dissipated as translation kinetic energy to all ions, based on the mass ratio of the Mg and O atoms. Therefore, the minimum kinetic energy spent in the electronic excitation is estimated to be 6 eV per four atoms. At a typical temperature for a Mg-O_2_ flame of 3300 K^28^, the thermal equilibrium condition gives a negligible ratio of molecules possessing the required kinetic energy. However, the energy released by an oxidation of a pair of Mg_2_ and O_2_ molecules is 12.4 eV^28^, which is sufficient to induce the high-energy collision of another pair of Mg_2_ and O_2_ under non-equilibrium conditions. This electronic excitation can cause light emission.

**Figure 3:**
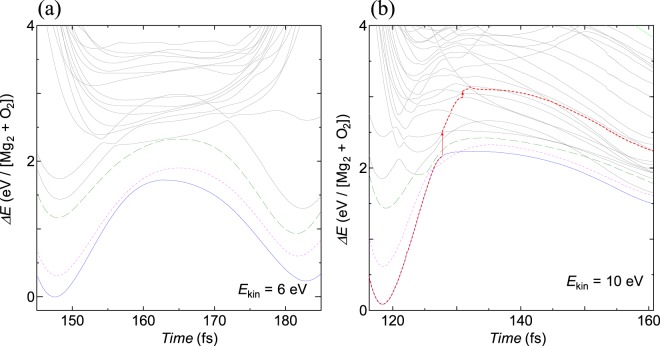
PESs landscape obtained by CASSCF calculations for triplet states for O_2_ and Mg_2_ along with the snapshot obtained by TDDFT Ehrenfest dynamics colliding with incident kinetic energies (*E*_*kin*_) of (**a**) 6 eV and (**b**) 10 eV. The lateral axis (*Time*) denotes the corresponding atomic coordinates of the TDDFT Ehrenfest dynamics. Ground, excited and next-excited states are shown in solid blue, dotted purple and dashed green lines, respectively. The arrows in (**b**) indicate possible upward transitions. Red dotted line in (**b**) indicates possible trajectory to an excited state. Note that with *E*_*kin*_ = 10 eV, the triplet ground state crosses with excited states, while the three states are well separated for *E*_*kin*_ = 6 eV.

We validated the PES landscape by performing CASSCF calculations with the same set of snapshots used throughout the TDDFT Ehrenfest dynamics. The multiple spin states, which were not included in the DFT ΔSCF calculations, were considered in the precise evaluation of the PESs. Figure 3 shows the triplet state landscape. When we followed the trajectory of the TDDFT Ehrenfest dynamics with an initial incident energy of 6 eV, the triplet ground state was well separated from the triplet excited states (Fig. 3(a)). Therefore, the electronic state was unlikely to transfer to the excited state in this case. When we followed the trajectory with a larger incident energy of 10 eV, the ground state was near several excited states. If a small upward transition occurred (Fig. 3(b)), the electronic state could be raised to higher excited states. To validate the currently used 6-31G basis set, we checked the PES landscape with 6-31G(d) basis set which includes polarization effect. The results shows the relative PES landscape is less sensitive to inclusion of the polarization, see the Supplementary Materials [See Supplementary Material at http://xxxx for numerical details.]. These behaviors of the PES landscape qualitatively explain the results of the TDDFT calculations despite the subtle difference in quantities.

**Figure 4:**
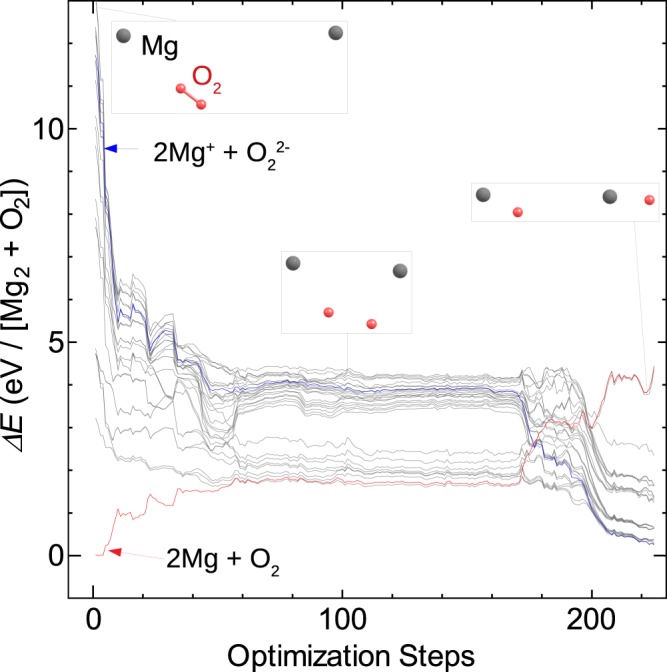
Evolution of triplet states during geometry optimization of triplet charge-transfer (CT) state of Mg_2_O_2_ calculated by state-averaged CASSCF. Crossing of neutral state (NS), which is originally the ground state, and CT state takes place around 180th step.

The current TDDFT-Ehrenfest dynamics and comparison with CASSCF suggest crossing of PES of the neutral state (NS) and CT states along with particular trajectory of MD. In order to examine the generality of the crossing, geometry optimization by CASSCF was performed on NS and CT, respectively, and the alternation between them was examined. Mg_2_ and O_2_ were rearranged to form two MgO flakes through dissociation of the O_2_ molecule (Fig. 4). During the rearrangement, the CT state and the NS intersected each other. In the final conformation, NS became an excited state situated about 4 eV above the CT state, which became the ground state. Thus, a high-energy collision exchanged the ground and excited states through reordering the atoms. The energy level of NS was raised by about 4.5 eV from the initial ground state. Next, we re-examined the collision of an O_2_ molecule with an Mg_2_ molecule. Classical kinetics tells us that about 40% of the incident kinetic energy of the O_2_ molecule dissipates into translational kinetic energy for all the ions. After the dissipation, the incident kinetic energy of 6 eV became equivalent to 3.6 eV available for electronic excitation, which was slightly lower than the energy difference of the NS for 2Mg^+^ + ${{\rm{O}}}_{2}^{2-}$ and Mg_2_ + O_2_. This fact is consistent with the observation that the incident kinetic energy of 6 eV was not enough for the transition to the PES with the lowest excitation energy.

### O_2_ collision with SiH_4_

**Figure 5:**
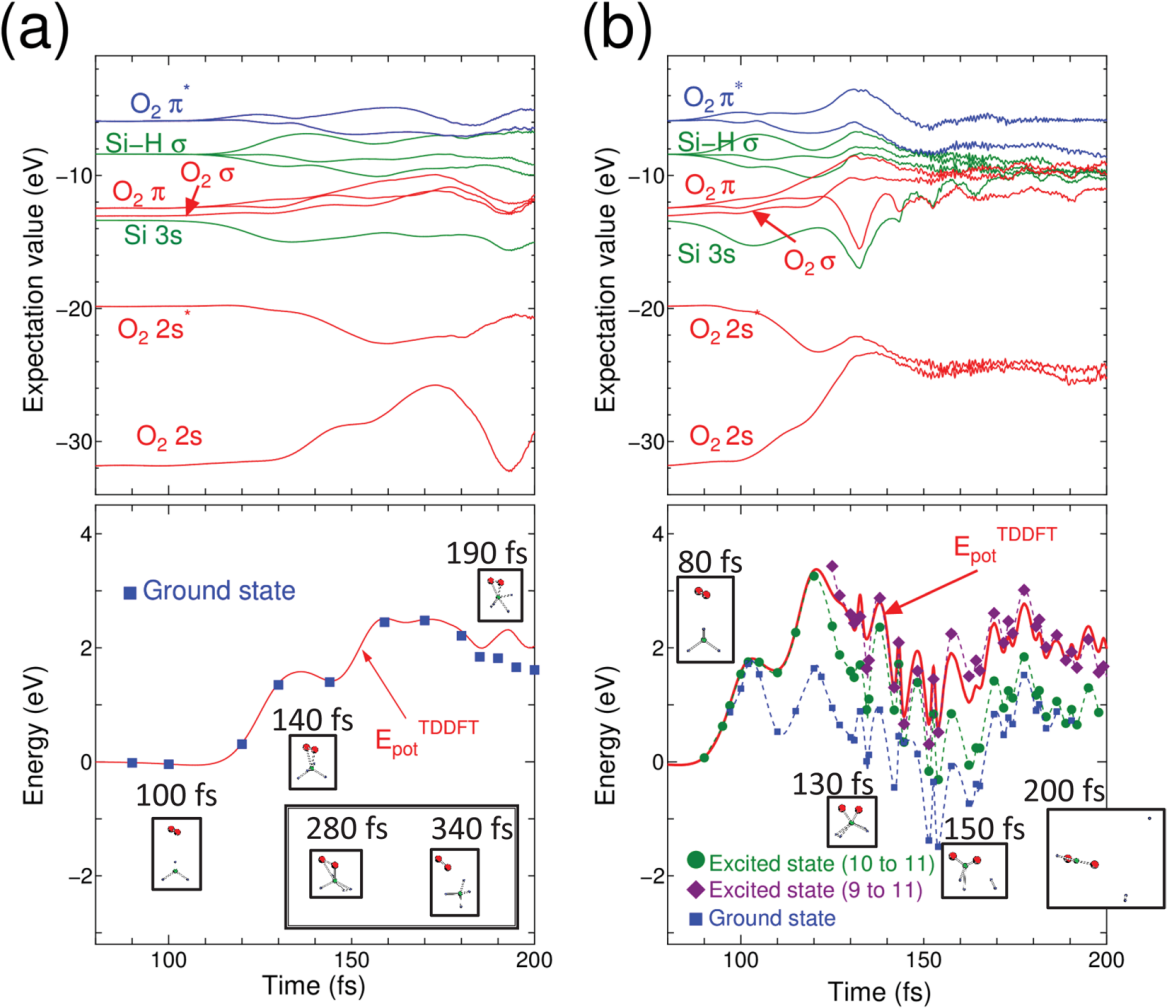
(**a**) (Top)Time evolution of the expectation values of molecular orbitals (MOs) for O_2_ colliding to SiH_4_ molecule with incident kinetic energy 6 eV. Red solid lines are for O_2_ fully occupied MOs, and blue solid lines are for O_2_ half occupied MOs. Green solid lines are for MOs of SiH_4_. (Bottom)Time-variation of the potential expressed as Eq. (1) of real-time TDDFT Ehrenfest dynamics. Potentials obtained by DFTground state calculation along with snapshots of TDDFT-Ehrenfest dynamics within the ΔSCF scheme are also shown as blue squares. Insets are atomic coordinates obtained by the TDDFT Ehrenfest dynamics of O_2_ (red colored balls) and SiH_4_ (Si atom as a green ball, and H atoms as blue small balls). Note two atomic coordinates beyond 200 fs are shown to express repulsion of the O_2_ molecule from the SiH_4_ molecule. (**b**) Same as (**a**) but with kinetic energy of 10 eV. In the bottom, the ΔSCF potential profiles on snapshot along with trajectory of TDDFT Ehrenfest dynamics are shown; Green circles denote excitation promoting one electron from 10th MO to 11th MO, while purple diamonds denote excitation promoting one electron from 9th MO to 11th MO, respectively.

We used initial incident energies of the O_2_ molecules of 4, 6, and 10 eV. In contrast to the O_2_ → Mg_2_ collision, oxidation was not observed for 4 and 6 eV, but it was observed for 10 eV. Figure 5(a,b) show the cases with incident kinetic energies of 6 and 10 eV, respectively. The top panels of Fig. 5(a,b) show the time evolution of the expectation values of the Kohn-Sham Hamiltonian for the MOs. There were 11 occupied MOs, seven of which originated from the O_2_ molecule and four of which originated from the SiH_4_ molecule. The highest levels were for the O_2_ HOMO, which was half-occupied. Thus, in the ground state, 10th and 11th orbitals were half-occupied. In contrast to the O_2_ → Mg_2_ collision case, the level alternation between the HOMO of the O_2_ molecule and MOs of the SiH_4_ molecule did not occur with an incident kinetic energy 6 eV. Yet we must note here again that alternation of the expectation values does not precisely mean the electronic excitation as discussed above.

**Figure 6:**
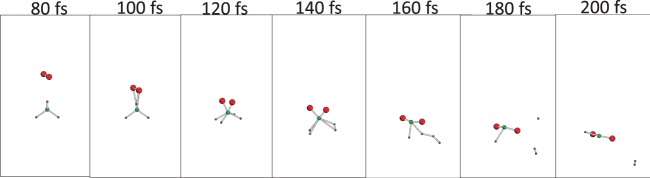
Snapshots of O_2_ colliding to SiH_4_ with incident kinetic energy of 10 eV. Red balls are O atoms, green medium sized balls are Si atoms, and small balls are H atoms.

The bottom panels of Fig. 5(a,b) show the time evolution of the TDDFT potential energy (Eq. (1)) throughout the TDDFT-Ehrenfest dynamics. The DFT (ΔSCF) potential was computed along with the snapshots of the trajectory of the TDDFT-Ehrenfest dynamics. For the incident energy of 6 eV, the DFT ground state potential almost agreed with that of the TDDFT-Ehrenfest dynamics up to 180 fs, and a tiny discrepancy was observed later. For the incident kinetic energy of 10 eV, the deviation of the potential energy between the TDDFT-Ehrenfest dynamics and ground state DFT calculation was large; thus, we examined the PESs of the excited states. The ΔSCF calculations of the excitation PESs were performed by assuming promotion of one electron from the 10th to 11th levels (green circles), and the promotion of one electron from the 9th to 11th levels (purple diamonds). The TDDFT potential merged with the ΔSCF results for the electron promotion from the 9th to 11th levels. Therefore, similar to the O_2_ → Mg_2_ collision, the electronic excitation and subsequent light emission was likely with an incident kinetic energy of 10 eV. The insets in Fig. 5 show the changes in atomic structure of the formation of two Si–O bonds after dissociation of the O_2_ molecule. For an incident kinetic energy of 6 eV, the O_2_ molecule was finally repelled from the SiH_4_ molecule. With an incident kinetic energy of 10 eV, a SiO_2_H_2_ molecule was formed. (Note that the snapshot of the TDDFT-Ehrenfest dynamics with incident energy of 10 eV is shown in Fig. 6).

We tried to perform the CASSCF calculation to validate this PES landscape. However, the active space with the frontier orbitals of the Si and O atoms was insufficient, even qualitatively, to describe the reaction. The present code could not handle an active space that included H 1 *s* orbitals. The CASSCF results for SiH_4_, with the combustion of other molecules, will be reported elsewhere.

### O_2_ collision with CH_4_

**Figure 7:**
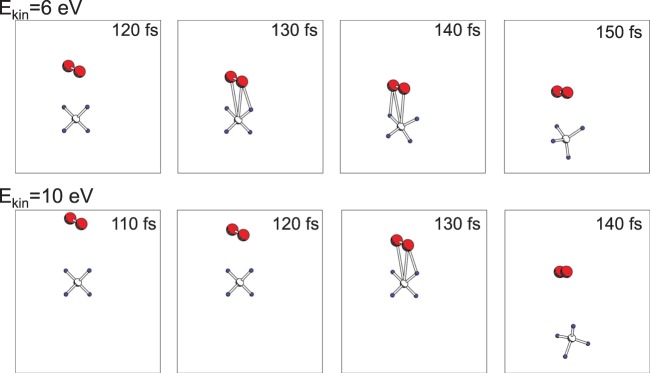
Snapshots of an O_2_ molecule colliding to a CH_4_ molecule with incident kinetic energy of 6 eV (top panels) and 10 eV (bottom panels). Red balls are O atoms, medium white balls are C atoms, and small balls are H atoms.

It is of fundamental interest to study a case of CH_4_ molecules which can be a counterpart of SiH_4_ as C is group-IV element like as Si. By using the scheme of TDDFT Ehrenfest dynamics, we collide an O_2_ molecule to a CH_4_ molecule with incident kinetic energies of 6 eV and 10 eV. Figure 7 shows the results demonstrating reflection of O_2_ and CH_4_ molecules instead of formation of C-O or C-H bonds. This is in sharp contrast to the case of O_2_ collision with SiH_4_, which may be because of smaller mass of CH_4_ than SiH_4_. From this result, we expect that collision of an O_2_ molecule with a molecule in the same order of mass to CH_4_ like as NH_3_ may also results in reflection instead of oxidation.

## Summary

In the current simulations, we monitored the electronic state that became an excited state upon oxidation. Several trajectories for the TDDFT-Ehrenfest dynamics of the collision of an O_2_ molecule with an incident kinetic energy of 4, 6, and 10 eV with target molecules (Mg_2_, SiH_4_, or CH_4_) were examined. For O_2_ → Mg_2_ collision, Mg–O bond formation was observed with an incident kinetic energy greater than 6 eV, and the electronic excitation energy increased with the incident kinetic energy. For the O_2_ → SiH_4_ collision, Si–O bond formation was observed at an incident kinetic energy of 10 eV. The mechanisms of electronic excitation were likely due to intrinsic nature of the PES landscape in which the PES of the ground state becomes closer to that of an excited state in some areas of the trajectories in the MD simulation. The PES landscape for the O_2_ → Mg_2_ collision was validated by CASSCF calculation, which was challenging for the O_2_ → SiH_4_ collision. Meantime, for O_2_ → CH_4_ collision with incident kinetic energy up to 10 eV, no oxidation reactions were observed, which may be due to smaller mass of CH_4_ than SiH_4_. From this fact, we do not expect the alternation of potential energy surface in collision of O_2_ molecules with small-mass molecules. We note here that studying oxidation of small-mass molecules, for instance CH_4_, NH_3_, and H_2_, are important subject of exploration of new fuel alternative to fossil fuel, however, they are out of the scope of current topic of light emission throughout the combustion.

## Supplementary information

Supplementary Material
