# Supplementary material for: Molecular-scale modeling of light emission by combustion: An ab initio study
Source: Sci Rep. 2019 Sep 3;9:12707. doi: 10.1038/s41598-019-49200-2 (PMC6722116; doi:10.1038/s41598-019-49200-2)
Supplement: Supplementary file 1 — Supplementary Material [file 41598_2019_49200_MOESM1_ESM.pdf]

Supplementary Material for ‘Molecular-scale modeling of light emission by combustion: An *ab initio* study’ by Yoshiyuki Miyamoto and Tokutaro Komatsu

The effects of the polarization function added to the basis function

For Mg_2 – O_2 collision with initial kinetic energy 10 eV, the calculated energies are shown in Figures S1 and S2. The inclusion of polarization function (6-31G(d)) improves the accuracy of the calculation by *ca*. 0.1 Hartree, however, the energy changes of each state are almost equal. Please note that in Figure 3, the energy surfaces, which are well separated at t = 116 fs, are closely situated with each other at t = 126 fs. As shown in Figure S2, the ‘squeezing’ of the states is clearly reproduced by 6-31G(d) basis set. Therefore, the conclusion is not affected by the choice of basis function, at least qualitatively.

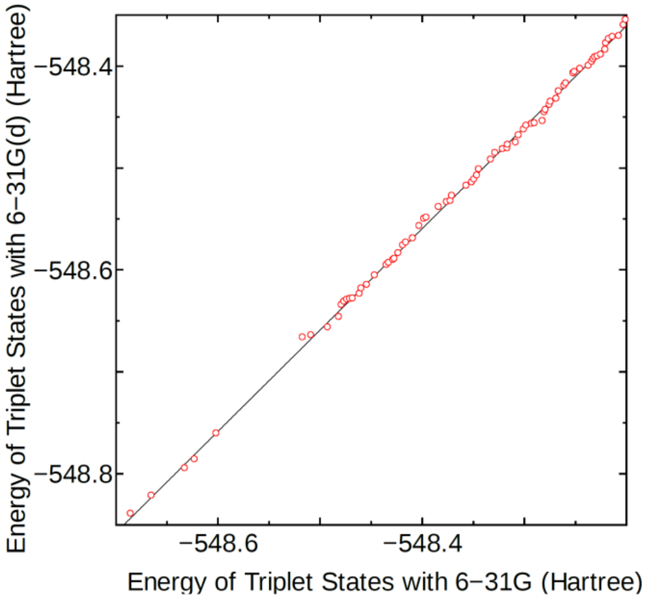

Figure S1: Comparison of the energies of triplet states at t = 116 fs calculated with modified 6-31G basis set and 6-31G(d) basis set.

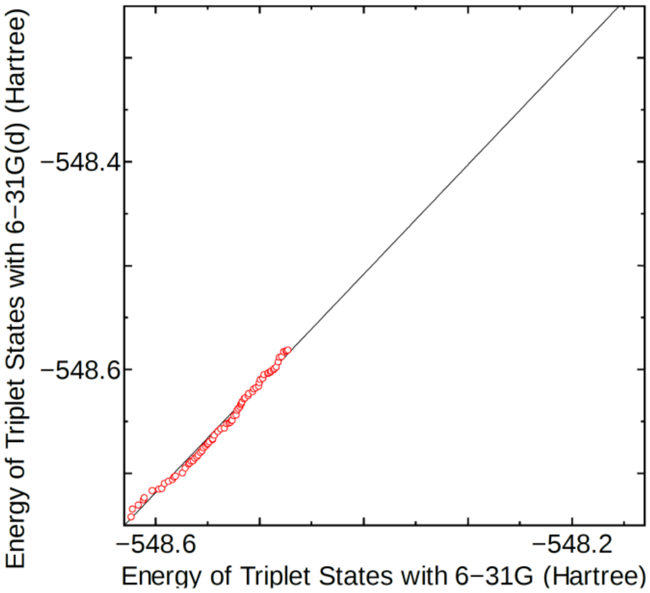

Figure S2: Comparison of the energies of triplet states at t = 126 fs calculated with modified 6-31G basis set and 6-31G(d) basis set.
